# Calcification of the acetabular labrum of the hip: prevalence in the general population and relation to hip articular cartilage and fibrocartilage degeneration

**DOI:** 10.1186/s13075-018-1595-y

**Published:** 2018-05-30

**Authors:** Thelonius Hawellek, Jan Hubert, Sandra Hischke, Matthias Krause, Jessica Bertrand, Burkhard C. Schmidt, Andreas Kronz, Klaus Püschel, Wolfgang Rüther, Andreas Niemeier

**Affiliations:** 1 Department of Orthopaedics, University Medical Center Hamburg-Eppendorf, Martinistraße 52, 20246 Hamburg, Germany; 2 Department of Medical Biometry and Epidemiology, University Medical Center Hamburg-Eppendorf, Hamburg, Germany; 3 Department of Osteology and Biomechanics, University Medical Center Hamburg-Eppendorf, Hamburg, Germany; 4 Department of Orthopaedic Surgery, Otto-von-Guerricke-University Magdeburg, Magdeburg, Germany; 5 Centrum of Geoscience, Georg-August-University Göttingen, Göttingen, Germany; 6 Department of Legal Medicine, University Medical Center Hamburg-Eppendorf, Hamburg, Germany

**Keywords:** Acetabular labrum, Hip, Chondrocalcinosis, Cartilage calcification, CPPD, Osteoarthritis

## Abstract

**Background:**

Meniscal calcification is considered to play a relevant role in the pathogenesis of osteoarthritis of the knee. Little is known about the biology of acetabular labral disease and its importance in hip pathology. Here, we analyze for the first time the calcification of the acetabular labrum of the hip (ALH) and its relation to hip cartilage degeneration.

**Methods:**

In this cross-sectional post-mortem study of an unselected sample of the general population, 170 ALH specimens and 170 femoral heads from 85 donors (38 female, 47 male; mean age 62.1 years) were analyzed by high-resolution digital contact radiography (DCR) and histological degeneration grade. The medial menisci (MM) from the same 85 donors served as an intra-individual reference for cartilage calcification (CC). Scanning electron microscopy (SEM), energy dispersive analysis (ED) and Raman spectroscopy were performed for characterization of ALH CC.

**Results:**

The prevalence of CC in the ALH was 100% and that in the articular cartilage of the hip (ACH) was 96.5%. Quantitative analysis revealed that the amount of ALH CC was higher than that in the ACH (factor 3.0, *p* < 0.001) and in the MM (factor 1.3, *p* < 0.001). There was significant correlation between the amount of CC in the fibrocartilage of the left and right ALH (*r* = 0.70, *p* < 0.001). Independent of age, the amount of ALH CC correlated with histological degeneration of the ALH (Krenn score) (*r* = 0.55; *p* < 0.001) and the ACH (Osteoarthritis Research Society International (OARSI), *r* = 0.69; *p* < 0.001). Calcification of the ALH was characterized as calcium pyrophosphate dihydrate deposition.

**Conclusion:**

The finding that ALH fibrocartilage is a strongly calcifying tissue is unexpected and novel. The fact that ALH calcification correlates with cartilage degeneration independent of age is suggestive of an important role of ALH calcification in osteoarthritis of the hip and renders it a potential target for the prevention and treatment of hip joint degeneration.

## Background

Osteoarthritis (OA) is a major health problem and represents the most common joint disease in western populations [1]. OA affects the whole joint and therefore involves hyaline cartilage, subchondral bone, the joint capsule, periarticular ligaments and fibrocartilage [2]. Until now the understanding of the molecular events that initiate and maintain OA pathogenesis remain incompletely understood. One factor of interest is cartilage calcification (CC). It is detectable in 100% of the hyaline cartilage of end-stage hip and knee OA [3, 4]. Calcium crystals have the potential to induce a pro-inflammatory intra-articular milieu [5–8] and also to alter the biomechanical properties of the cartilage [9, 10], both of which may finally result in OA [11].

Compared to articular hyaline cartilage, the fibrocartilage of the meniscus of the knee seems to be particularly prone to calcification [12, 13] and meniscal calcification is highly prevalent in knee OA [14, 15]. In addition, meniscal cells calcify more readily in OA than in healthy knees and calcification may alter the biomechanical properties of the meniscus, which may further contribute to OA development [14]. Accordingly, meniscal calcification is considered to play a relevant role in the pathogenesis of knee OA [14–18].

In contrast to the meniscus of the knee, little is known about calcification of the fibrocartilage of the hip, the acetabular labrum (ALH) and its relation to cartilage degeneration. Although the role of the acetabular labrum in hip joint pathology has recently gained much attention [19, 20], knowledge about cellular mechanisms that govern labral function is scarce. It has recently been described that ALH cells appear to have a similar metabolic profile to meniscal cells [21], but to our knowledge there is only one study in which ALH calcification was analyzed in a larger cohort (106 hip joints in 66 individuals) by computed tomography (CT), finding a prevalence of 18% [22]. Of note, CC starts in the nanomicrometer to micrometer range and is therefore hard or almost impossible to detect in the initial stages by standard radiographic methods with low-resolution x-ray, CT or magnetic resonance imaging (MRI). Therefore, little is known about the actual prevalence of early initial crystallization in human joints in general, and in the ALH in particular [23]. The most sensitive method available for the detection of such micro-calcifications is high-resolution digital contact radiography (DCR) [4, 24]. A disadvantage of this method is that DCR can only be applied to tissue samples ex vivo.

The goal of the present study was to describe the prevalence of DCR-detectable ALH calcification in an unselected sample of the general population and to analyze the relationship between the amount of ALH calcification and the degree of fibrocartilage and articular cartilage degeneration in the hip. Since we have recently described that there appears to be a systemic drive for CC [25, 26], here we used both medial menisci (MM) as a reference for the individual propensity to develop CC.

## Methods

A total of 170 ALH, femoral heads (FH) and MM were obtained from both hip and knee joints in an unselected sample of 85 individuals (hereafter referred to as “donors”) who underwent autopsy at the Department for Legal Medicine [27], University Medical Center Hamburg-Eppendorf. Only donors with bilaterally intact hip and knee joints without any signs of hip and/or knee disease other than OA were included in this study. None of the donors had evidence of previous hip and/or knee surgery. Donors with history of tumors, infections or rheumatic diseases were excluded from the study population. The study was approved by the local ethics committee (reference number PV4570) and is in compliance with the Helsinki Declaration. The mean age was 62.1 years (range 20–93 years); 38 of the donors were female and 47 male. Biometric characteristics of the donors are listed in Table 1. Some of the data on hip articular cartilage calcification in this larger cohort have previously been analyzed and published in another context [25]. First the FH, ALH and MM were resected in toto. Any attached soft tissue was removed from the ALH, FH and MM. For FH analysis, standardized 4 mm bone and cartilage slabs were cut in the central coronal and axial planes, resulting in three standardized (central, anterior and posterior) slabs per sample as published previously [25]. ALH and MM were kept in toto.

**Table 1:** Biometric characteristics of the study population (*n* = 85)

Characteristic	Value
Age in years	62.1 ± 19.3
Male	60.1 ± 18.6
Female	64.6 ± 20.0
Height in cm	
Male	176.9 ± 7.1
Female	164.7 ± 7.9
Body weight in kg	
Male	83.2 ± 18.3
Female	72.3 ± 21.0
Body mass index in kg/m^2^	26.5 ± 6.0

### Digital contact radiography (DCR)

The ALH, the bone-cartilage slabs of the FH and the MM were washed with physiological solution to remove residual bone debris. Standardized radiographs were taken (25 kV, 3.8 mAs, film focus distance 8 cm) using a high-resolution digital radiography device (Faxitron X-Ray, Illinois, USA). Quantitative computerized analysis of the areas of CC of each complete ALH, the three bone-cartilage slabs of the FH and the complete MM was performed with standard software (ImageJ 1.46, National Institutes of Health, Bethesda, USA) as published previously [3, 4, 28]. The percentage of calcification of the ALH and MM was determined by dividing the measured area of calcification by the total fibrocartilage area of the particular anatomical structure. The percentage of CC in each of the three slabs of the FH was determined by dividing the measured area of calcification by the total cartilage area per slab. The mean amount of calcification measured from the three slabs of the FH was regarded to be representative of the entire articular cartilage of the hip (ACH).

### Classification of acetabular labrum calcification

**Fig. 1:**
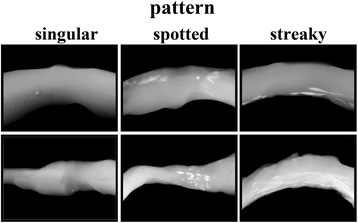
Exemplary samples of digital contact radiography images of the acetabular labrum from six different donors with distinct cartilage calcification. Cartilage calcification was detected as radiopaque spots within the fibrocartilage of the acetabular labrum. The cartilage calcification was classified into three typical calcification patterns (singular, spotted and streaky)

Based on previously published soft tissue classifications [29–31], the distribution of ALH calcification was categorized as three different patterns (singular, spotted or streaky) on DCR images (Fig. 1).

### Histology

**Fig. 2:**
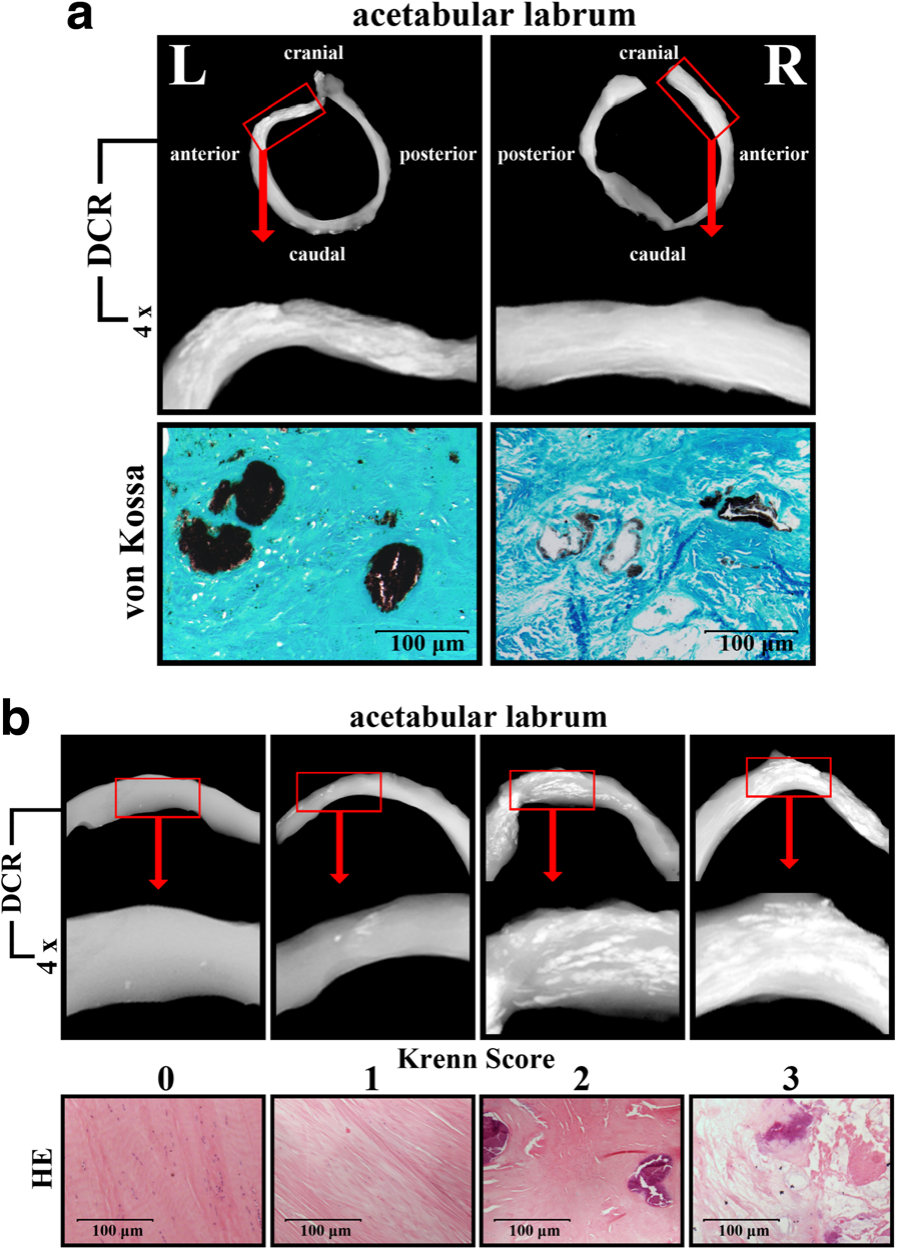
**a** Representative digital contrast radiography (DCR) images (presented in original size and × 4 magnification (red boxes)) of the acetabular labrum (L = left, R = right) from one donor showing distinct cartilage calcification and the corresponding histological images in which cartilage calcifications (black) were confirmed histochemically by von Kossa staining. **b** DCR images (presented in original size and × 4 magnification (red boxes)) with increasing cartilage calcifications (from left to the right) of the acetabular labrum from different donors and the corresponding rising histological degeneration grade, which was evaluated by the Krenn score (0–3) on hematoxylin-eosin (HE) staining

Histological degeneration of the fibrocartilage of the superior-anterior part of each ALH and of the hyaline cartilage of the main load-bearing zone of each FH (central zone, directly adjacent to the central slab plane) was assessed. All specimens were fixed in 4% paraformaldehyde (PFA) for 24 h, dehydrated in 80% alcohol, embedded in paraffin and 4-μm sections were prepared. Sections of the ALH were stained with hematoxylin and eosin (Fig. 2b) and samples of the FH were stained with 1% Safranin-O to evaluate the histological degeneration grade of the tissue sample according to the Krenn-score for fibrocartilage (grade 0–3; Table 2) [32] and the Osteoarthritis Research Society International (OARSI) osteoarthritis cartilage histopathology assessment system for hyaline cartilage (grade 0–6) [33]. Calcifications identified by DCR were confirmed to represent calcium-phosphate crystal deposition by von Kossa staining (Fig. 2a).

**Table 2:** Krenn score – histopathological evaluation of the degeneration grade of the fibrocartilage (0–3)

Grade	Features
0	Normal histological morphology • Isomorphic chondrocytes • Homogeneously eosinophil-stained matrix • Regular cellularity
1	Low-grade degeneration • Low reduction of cellularity (small areas) • Inhomogeneous stained matrix • Small fissures in the matrix
2	Moderate degeneration • Moderate reduction of cellularity (large areas) • Variable size and shape of chondrocytes • Moderate fissures in the matrix
3	High-grade degeneration • Strong reduction of cellularity • Large areas of complete loss of chondrocytes • Reticular/basophilic stained matrix (mucoid degeneration) • Large fissures in the matrix (pseudocysts)

### Characterization of acetabular labrum calcification

To characterize the physicochemical nature of ALH CC detected by DCR and confirmed by von Kossa staining, 10 randomly selected labral specimens were processed for further analysis.

#### Scanning electron microscopy

For morphology studies semiquantitative electron probe microanalysis and scanning electron microscopy (SEM) of secondary electrons (SE) were performed on a JEOL 8900RL (JEOL, Ltd., Akishima, Japan) to obtain information about the surface of the ALH cartilage and minerals. The accelerating voltage was set to 10 kV and a beam current of 1.5 nA was used for the quantitative analysis. SEM images were performed at 10 kV accelerating voltage and a variable beam current between 0.1 and 0.25 nA.

To determine the chemical elements in the samples and to analyze the qualitative and semiquantitative chemical compositions raw x-ray counts were acquired by a SiriusSD® (SGX Sensortech Ltd.) energy dispersive (ED) silicon drift detector. For calibration a natural apatite crystal (Ca_5_(OH,Cl,F)(PO_4_)_3_) was used for calcium and phosphate and albite (NaAlSi_3_O_8_) was used for sodium. Prior to the measurement the samples were coated with a thin layer of gold (20 nm).

#### Raman spectroscopy

Raman spectroscopy was performed on unstained labrum specimens. Raman spectra were obtained using a Horiba Jobin Yvon HR 800 UV Raman spectrometer with an attached Olympus BX41 microscope. The samples were excited using a 488 nm laser line of a Coherent Sapphire solid-state laser, with 50 mW at the laser exit. The use of a holographic grating with 600 lines/mm and a CCD-detector with 1024 × 256 pixels yielded a spectral dispersion of better than 2.2/cm per pixel. Raman spectra were collected in three spectral windows in the range 200/cm to 4000/cm with an acquisition time of 2 × 2 s for each spectral window. The Raman spectra were frequency corrected using silicon (Si band at 520.4/cm), which was measured directly after the sample measurement. The collected Raman spectra were compared to reference spectra for hydroxyapatite (960 cm^− 1^) and calcium pyrophosphate dihydrate (1050 cm^− 1^) [34].

### Statistical analysis

The biometric characteristics of donors are reported as mean values ± standard deviations. For descriptive analysis, mean cartilage calcification values for the hyaline cartilage of the femoral head were used. Data were logarithmically transformed if appropriate. For categorical data Fisher’s and McNemar’s tests were used. A linear mixed model was used to analyze the difference between the mean amount of cartilage calcification in the ALH, FH and MM considering side and joint as fixed effects. Subject was used as a random effect with a compound symmetry covariance structure. In addition, the mixed model assumptions were checked using residual plots. To report the association between continuous variables Pearson’s (*r*) or Spearman’s (*r*_s_) rank correlation coefficient was calculated. To test the correlation between cartilage calcification, histological degeneration and age, the mean value of the left and right femoral head and the mean value of the left and right labrum were calculated for each individual. To avoid spurious correlation, a test of partial correlation was performed adjusting for the respective excluded parameters (cartilage calcification, histological OA grade and age). All statistical analyses were performed with statistical software R [35], version 3.1.1. *P* values less than 0.05 were considered statistically significant.

## Results

### Prevalence of cartilage calcification

The prevalence of CC of the ALH was 100% (85/85) (95% CI 0.96, 1.00), of the ACH it was 96.5% (82/85) (95% CI 0.90, 0.99) and of the MM it was 98.8% (84/85) (95% CI 0.94, 1.00) (Table 3). The prevalence of bilateral CC of the ALH was 100% (85/85) (95% CI 0.96, 1.00), of the ACH it was 80.0% (68/85) (95% CI 0.70, 0.88) and of the MM it was 92.9% (79/85) (95% CI 0.85, 0.97) (Table 3). Von Kossa stained histological sections confirmed that DCR-detectable CC actually represents calcium-phosphate crystal depositions at the histological level (Fig. 2a). CC was detected in 100% of the left and right ALH (85/85), in 88.2% of the left and right ACH (75/85), in 94.1% of the left MM (80/85) and in 97.6% of the right MM (83/85). There was no significant preponderance of CC according to left or right side in the ALH (*p* = 1.0), ACH (*p* = 1.0) and MM (*p* = 0.38) (Table 3). There was no significant difference in the prevalence of CC by sex in the ALH (*p* = 1.0), FH (*p* = 0.09) and MM (*p* = 1.0).

**Table 3:** Prevalence of DCR-detectable cartilage calcification (n = 85)

	Acetabular labrum		Femoral head		Medial meniscus	
	Number	Percentage	Number	Percentage	Number	Percentage
Total calcified cartilage (CC)	85/85	100	82/85	96.5	84/85	98.8
Bilateral CC	85/85	100	68/85	80.0	79/85	92.9
Unilateral CC	0/85	0	14/85	16.5	5/85	5.9
Left CC	85/85	100	75/85	88.2	80/85	94.1
Right CC	85/85	100	75/85	88.2	83/85	97.7

### Quantitative analysis of ALH calcification reveals a higher degree of calcification than in articular and meniscal cartilage

**Fig. 3:**
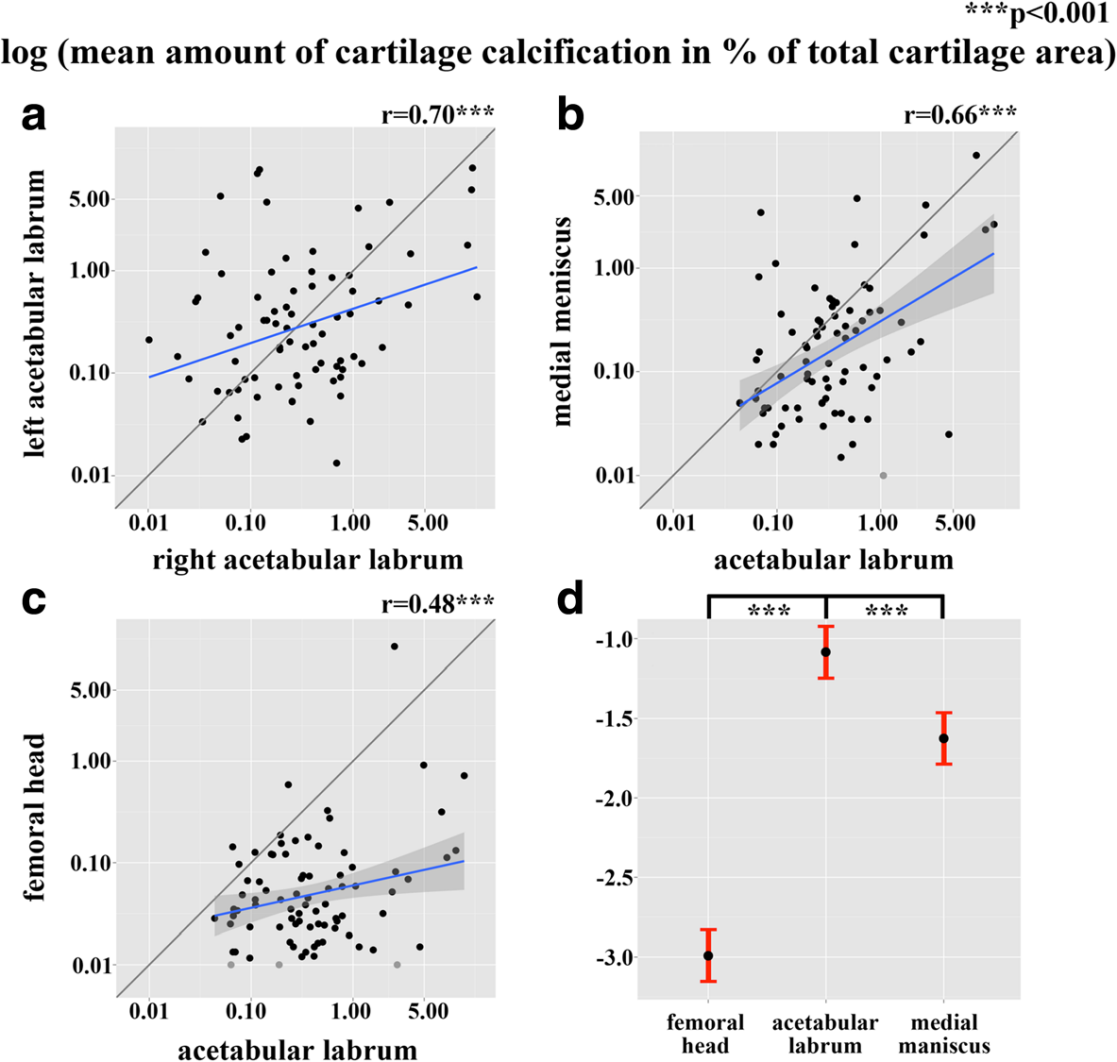
**a**–**c** Logarithmic scatter plots show significant correlation for the mean amount of calcified cartilage (CC) in percentage of total cartilage area between the left and right acetabular labrum (*r* = 0.70, 95% CI 0.57 0.79), *p* < 0.001) (**a**), the medial meniscus and the acetabular labrum (*r* = 0.66, 95% CI 0.52 0.76), *p* < 0.001) (**b**) and the femoral head and the acetabular labrum (*r* = 0.48, 95% CI 0.30 0.63), *p* < 0.001) (**c**). Data points are jittered to avoid over plotting. Logarithmic scatter plots are shown with the blue orthogonal regression line and with the corresponding correlation coefficient (*r*). **d** Logarithmic effect plot of the mean amount of CC in percentage of total cartilage area for the femoral head, the acetabular labrum and the medial meniscus. The amount of CC in the fibrocartilage of the acetabular labrum was significant larger compared to the amount of CC in the medial meniscus (factor of 1.3, *p* < 0.001) and the amount of CC in the acetabular labrum was significant larger compared to the amount of CC in the hyaline cartilage of the femoral head (factor of 3.0, *p* < 0.001)

There was significant correlation between the amount of CC in the fibrocartilage of the left and right ALH (*r* = 0.70, *p* < 0.001, 95% CI 0.57 0.79) (Fig. 3a) and between the ALH and the MM (*r* = 0.66, *p* < 0.001, 95% CI 0.52 0.76) (Fig. 3b) and the ALH and the ACH (*r* = 0.48, *p* < 0.001, 95% CI 0.30 0.63) (Fig. 3c).

A significant difference was found between the amount of CC in the three distinct cartilage tissues, with the amount of CC in the fibrocartilage in the ALH being significantly higher than that in the ACH by factor 3.0 (*p* < 0.001) and higher than that in the MM by factor 1.3 (*p* < 0.001) per tissue volume unit (Fig. 3d).

### Calcification of the ALH correlates with histological hip degeneration independent of age

**Fig. 4:**
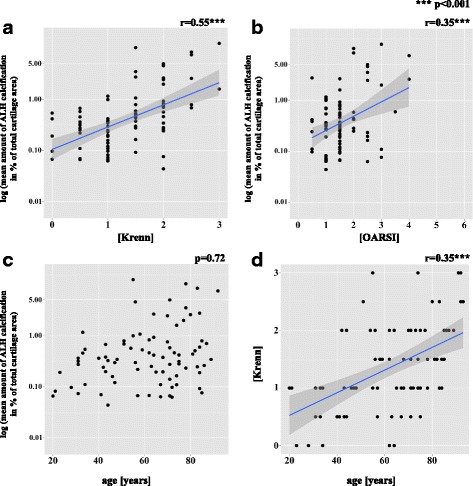
Logarithmic scatter plots show significant correlation between the mean amount of calcification (percentage of total cartilage area) of the acetabular labrum of the hip (ALH) and the histological degeneration grade (Krenn) of the ALH (*r* = 0.55, *p* < 0.001) after adjustment for age (**a**), the mean amount of ALH calcification (percentage of total cartilage area) and the histological osteoarthritis (OA) grade for the hyaline cartilage (Osteoarthritis Research Society International (OARSI)) of the femoral head (*r* = 0.35, *p* < 0.001) after adjustment for age (**b**) and the histological degeneration grade (Krenn) of the ALH and age (*r* = 0.35, *p* < 0.001) after adjusting for cartilage calcification (**d**). **c** There was no correlation between the mean amount of ALH calcification (percentage of total cartilage area) and age (*p* = 0.72) after adjusting for the histological degeneration grade. Data points are jittered to avoid over plotting. Logarithmic scatter plots are shown with the blue orthogonal regression line and with the corresponding correlation coefficient (*r*)

When adjusted for age there was significant correlation between the amount of CC in the ALH and the histological degeneration grade of the ALH according to the Krenn score (*r* = 0.55, *p* < 0.001) (Figs. 2b and 4a). The distribution of the degeneration grade with detailed data on the donors and according to the mean amount of CC in the ALH is shown in Table 4. Moreover, there was significant correlation between the amount of CC in the ALH and the histological OA grade of the ACH (OARSI) after adjustment for age (*r* = 0.35, *p* < 0.001) (Fig. 4b). There was significant correlation between the amount of CC in the ALH and age (r_s_ = 0.32, *p* = 0.003), which was no longer significant after adjustment for the histological ALH degeneration grade (*r*_s_ = 0.04, *p* = 0.72) (Fig. 4c). There was significant correlation between the histological degeneration grade of the ALH and age (*r* = 0.50, *p* < 0.001), which persisted after adjustment for CC (*r* = 0.35, *p* < 0.001) (Fig. 4d).

**Table 4:** Distribution of the degeneration grade of the acetabular labrum (n = 170)

Grade	Number	Percentage	Male	Percentage	Female	Percentage	Mean amount of calcified cartilage
0	26/170	15.3	17/94	18.1	9/76	11.8	0.25 (SD ±0.31)
1	79/170	46.5	37/94	39.4	42/76	55.3	0.37 (SD ±0.77)
2	44/170	25.9	28/94	29.8	16/76	21.1	1.69 (SD ±4.40)
3	19/170	11.2	10/94	10.6	9/76	11.8	11.70 (SD ±12.25)

### Classification of acetabular labrum calcification

In 56.5% of all analyzed samples calcification could be classified as a singular calcification pattern. In 28.8% and 14.7% of the samples, respectively, a spotted or a streaky calcification pattern was detected (Fig. 1). In samples with a singular calcification pattern the mean amount of calcification was 0.38% (SD ±0.68) and the mean histological degeneration grade was 1.1 (SD ±0.8). In samples with a spotted or a streaky calcification pattern, respectively, the mean amount of calcification was 0.91% (SD ±1.54) and 10.01% (SD ±12.2) and the mean histological degeneration grade was 1.5 (SD ±0.9) and 2.0 (SD ±1.0) (Table 5).

**Table 5:** Calcification pattern of the acetabular labrum (n = 170) with the corresponding mean histological degeneration grade (Krenn score) and the mean amount of cartilage calcification

Pattern			Krenn Score		Mean calcified cartilage	
	Number	Percentage	Mean	SD	Percentage	SD
Singular	96/170	56.5%	1.1	±0.8	0.38	±0.68
Spotted	49/170	28.8	1.5	±0.9	0.91	±1.54
Streaky	25/170	14.7	2.0	±1.0	10.01	±12.2

### Characterization of acetabular labrum calcification

**Fig. 5:**
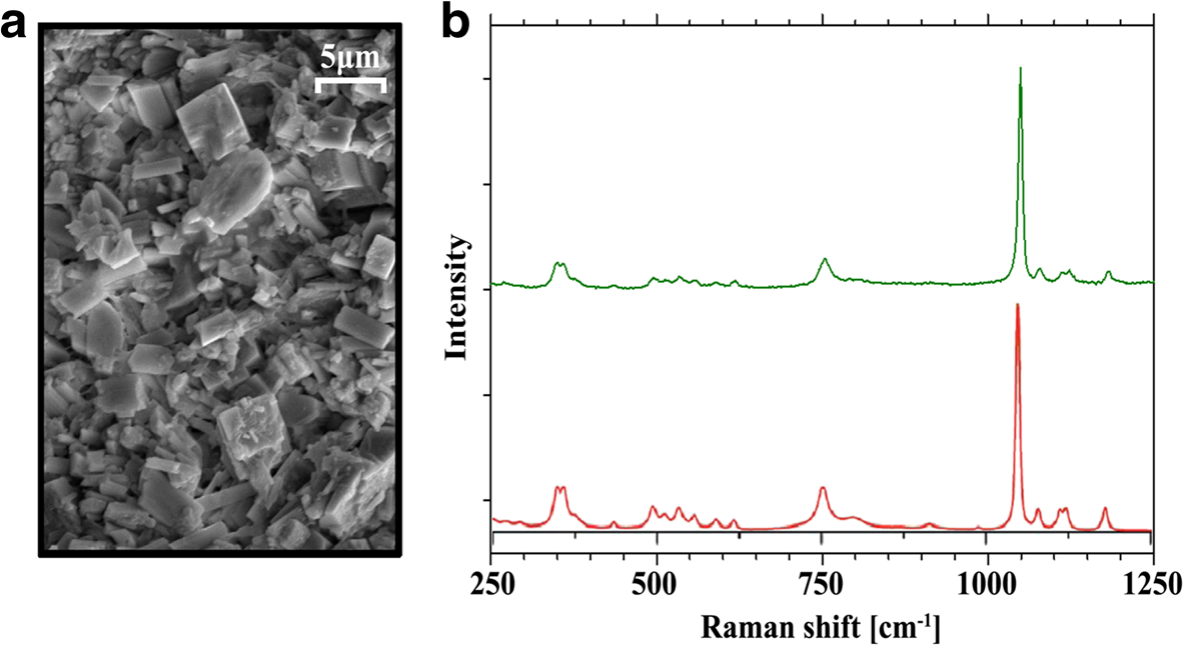
**a** Exemplary sample of scanning electron microscopy (SEM) imaging of the acetabular labrum of the hip (ALH) fibrocartilage showing well-developed rhomboid and prismatic crystals in various sizes and spatial arrangements, indicating the presence of calcium pyrophosphate dihydrate (CPPD) crystals. **b** Corresponding sample of measured Raman spectra (green line) with a peak count at 1050 cm^− 1^ confirming the presence of CPPD crystals in comparison to the reference spectra of t-CPPD (red line)

Well-developed crystals of prismatic and rhomboid habit, the typical form of calcium pyrophosphate dihydrate (CPPD) crystals, were detected by SEM imaging (Fig. 5a). Using energy dispersive qualitative element analysis (ED) oxygen, phosphorous, calcium and small amounts of sodium were detected. The mean calcium/phosphate (Ca/P) molar ratio was approximately 1.0 in all samples, indicating the presence of CPPD crystals. By Raman spectroscopy only spectra could be detected, which can be assigned to triclinic calcium pyrophosphate dihydrate (t-CPPD, Ca_2_P_2_O_7_ · 2H_2_O) (Fig. 5b). In conclusion, when assessed by SEM, ED and Raman spectroscopy, only CPPD crystals were detected in the ALH samples. We did not find evidence of the appearance of basic calcium phosphate crystals in the analyzed samples.

## Discussion

Here we demonstrated that fibrocartilage calcification of the ALH is highly prevalent in an unselected sample of the general population, including in histologically healthy tissue. The amount of ALH calcification correlates significantly with fibrocartilage and articular cartilage degeneration of the hip independent of age.

These data are entirely novel and shed new and unexpected light on the potential clinical relevance of ALH calcification. Given that in the existing literature the prevalence of ALH calcification is estimated to be smaller than 20% [22], the prevalence of 100% reported here was highly unexpected. This first-sight discrepancy is likely to be explained by the unequalled sensitivity of CC detection by DCR as compared to native CT [24]. Taking this into account, it was still striking to observe that both the prevalence and the amount of CC per unit of tissue volume were significantly higher in the ALH than in the ACH or the MM within the same individuals (Fig. 3d). Most interestingly in this context, we observed CC in histologically healthy labral tissue. Thus, labral calcification cannot just be a result or byproduct of degenerative tissue changes, but rather seems to precede the degeneration. In light of the correlation between the amount of CC in the ALH and histological degeneration of both the labrum and the articular cartilage independent of age (Fig. 4), these data open the possibility (although speculative at the present time) that the calcification crystals may be involved as a causative factor. We have previously reported that articular cartilage calcification can be looked at as the result of a systemically driven process [25, 26], which was reconfirmed by the present data, exemplified by correlation between the amount of CC in the ALH and that in the MM and the contralateral ALH in this unselected cohort of donors (Fig. 3a, b).

The results of this study support the idea that if the degree of CC in the ALH even in young individuals exceeds some yet to be defined threshold, this may trigger labral and subsequently total hip joint pathologic change. Further research will be needed to support this concept, in particular in regard to the underlying cellular and molecular mechanisms and in regard to the epidemiology of the degree of CC in early symptomatic labral tissue in young adults without the presence of advanced OA.

To further analyze the ALH calcification we developed a classification referring to previously published classifications of other types of soft tissue calcification [29–31]. By using three calcification patterns (single, spotted and streaky), we observed that streaky calcification is the pattern in ALH calcification with the highest mean amount of CC. Samples with a singular calcification pattern had the lowest mean amount of CC in ALH calcification. Samples with a spotted calcification pattern had a mean amount of CC that was between the other two patterns. Interestingly ALH samples with a streaky calcification pattern had more histological evidence of degeneration and samples with a singular calcification pattern had less degeneration on average. Moreover, samples with a spotted calcification pattern displayed moderate degeneration. To further characterize ALH calcification we performed scanning electron microscopy, energy dispersive analysis and Raman spectroscopy. By these analyses we found evidence for the deposition of calcium pyrophosphate dihydrate (CPPD) crystals only, but not of basic calcium phosphate (BCP). Several studies have characterized the calcification of hyaline articular cartilage in the hip [3] and knee [4, 36–39] joints. In these studies the detection of BCP and CPPD crystals was reported. To our knowledge there are only few studies in which calcification in the meniscus has been characterized [39, 40] and there are no studies available that have characterized calcification in the ALH. Using Fourier transform infrared (FTIR) spectroscopy, Dessombz et al. detected CPPD crystals in one human meniscus (fibrocartilage) while CPPD crystals and carbonated apatite were detectable at the same time in the other meniscus that was analyzed [39]. Kiraly et al. analyzed the type of crystals in 10 menisci by histological examination [40]. They reported that 80% of the calcified meniscal tissue contained CPPD crystals and 20% BCP crystals. They noticed that both crystal types can be found in the meniscal tissue but the large amount of crystals within the fibrocartilage of the knee appeared to be CPPD crystals. Currently, it remains speculative whether BCP crystals can be found in ALH calcification. In our ALH samples that underwent detailed physico-chemical crystal characterization (n = 10), we found only CPPD and no BCP crystals. We conclude that calcification of the ALH seems to appear mainly by CPPD deposition, but this need to be confirmed in future studies.

Limitations of the present study include limited available information on the medical history of the donors. Moreover there was no information about clinical symptoms of hip or knee pain and function. The standardized slab specimens of the FH reflect representative standardized planes, but only a small part of the articulating surface of the joint in absolute terms, which, theoretically, opens up the possibility of sampling error. None of the mentioned limitations is likely to have had any profound impact on the major new findings and conclusions that we draw from the present study.

## Conclusions

Calcification of the acetabular labrum of the hip is unexpectedly highly prevalent and occurs even in healthy labral tissue, but the amount of labral calcification significantly correlates with overall hip joint degeneration independent of age. Calcification of the acetabular labrum can be classified into three typical patterns (singular, spotted and streaky) and is mainly induced by calcium pyrophosphate dihydrate deposition. We propose that acetabular labral calcification deserves further detailed study as a potentially causative factor in labral pathological change and early osteoarthritis of the hip.

## Abbreviations

ACH: Articular cartilage of the hip
ALH: Acetabular labrum of the hip
BCP: Basic calcium phosphate
CC: Cartilage calcification
CT: Computed tomography
CPPD: Calcium pyrophosphate dihydrate
DCR: High-resolution digital contact radiography
ED: Energy dispersive analysis
FH: Femoral head
MM: Medial meniscus
OA: Osteoarthritis
OARSI: Osteoarthritis Research Society International
SEM: Scanning electron microscopy
